# Yellow loosestrife (*Lysimachia vulgaris* var. *davurica*) ameliorates liver fibrosis in *db/db* mice with methionine- and choline-deficient diet-induced nonalcoholic steatohepatitis

**DOI:** 10.1186/s12906-021-03212-6

**Published:** 2021-01-25

**Authors:** Yang-Ju Son, Da Seul Jung, Ji Min Shin, Myungsuk Kim, Gyhye Yoo, Chu Won Nho

**Affiliations:** Smart Farm Research Center, Korea Institute of Science and Technology (KIST), Gangneung Institute of Natural Products, Gangneung, Gangwon-do 25451 South Korea

**Keywords:** Yellow loosestrife, *Lysimachia vulgaris* var. *davurica*, Nonalcoholic fatty liver disease, Nonalcoholic steatohepatitis, Liver fibrosis, TGFβ/Smad signaling

## Abstract

**Background:**

Nonalcoholic steatohepatitis (NASH), a liver disease caused by a nonalcoholic fatty liver, is increasing in incidence worldwide. Owing to the complexity of its pathogenic mechanisms, there are no therapeutic agents for this disease yet. The ideal drug for NASH needs to concurrently decrease hepatic lipid accumulation and exert anti-inflammatory, antifibrotic, and antioxidative effects in the liver. Because of their multipurpose therapeutic effects, we considered that medicinal herbs are suitable for treating patients with NASH.

**Methods:**

We determined the efficacy of the alcoholic extract of *Lysimachia vulgaris* var. *davurica* (LV), an edible medicinal herb, for NASH treatment. For inducing NASH, C57BLKS/J lar-Lepr^db^/Lepr^db^ (*db*/*db*) male mice were fed with a methionine-choline deficient (MCD) diet ad libitum. After 3 weeks, the LV extract and a positive control (GFT505) were administered to mice by oral gavage for 3 weeks with a continued MCD diet as needed.

**Results:**

In mice with diet-induced NASH, the LV extract could relieve the disease symptoms; that is, the extract ameliorated hepatic lipid accumulation and also showed antioxidative and anti-inflammatory effects. The LV extract also activated nuclear factor E2-related factor 2 (Nrf2) expression, leading to the upregulation of antioxidants and detoxification signaling. Moreover, the extract presented remarkable efficacy in alleviating liver fibrosis compared with GFT505. This difference was caused by significant LV extract-mediated reduction in the mRNA expression of fibrotic genes like the alpha-smooth muscle actin and collagen type 3 alpha 1. Reduction of fibrotic genes may thus relate with the downregulation of transforming growth factor beta (TGFβ)/Smad signaling by LV extract administration.

**Conclusions:**

Lipid accumulation and inflammatory responses in the liver were alleviated by feeding LV extract to NASH-induced mice. Moreover, the LV extract strongly prevented liver fibrosis by blocking TGFβ/Smad signaling. Hence, LV showed sufficient potency for use as a therapeutic agent against NASH.

**Supplementary Information:**

The online version contains supplementary material available at 10.1186/s12906-021-03212-6.

## Background

Nonalcoholic fatty liver disease (NAFLD) is increasingly becoming a worldwide health issue owing to the burgeoning cases of obesity in the global population. In developed countries, almost one third of adults have NAFLD, and its incidence among adolescents is also increasing [1]. NAFLD is an umbrella term that represents diseases ranging from fatty liver to nonalcoholic steatohepatitis (NASH), where the disorder can progress to cirrhosis and liver cancer [2]. NAFLD onset is caused by lipid droplet accumulation in the liver, which is mainly attributed to excess food consumption because the liver is a primary organ of de novo lipid synthesis. Hepatocytes produce triglycerides (TGs) and release lipids to the portal vein in the form of very-low-density lipoproteins (VLDLs) [3]. Although NASH development has been associated with inflammation caused by oxidative stress from excess lipids stored in the liver, the underlying mechanisms of fatty liver remain to be solved [4, 5]. NASH can be distinguished from fatty liver based on the signs of inflammation, which can be accompanied with mild fibrosis. Continuous inflammatory reactions cause the loss of severely damaged hepatocytes, which are then superseded with fibrocytes [6]. Patients with cirrhosis, a form of NASH characterized by liver deterioration, experience irreversible liver dysfunction.

Development of efficacious therapies for NASH is still faced with unresolved challenges, owing to the multiple potential pathogenic pathways involved in the disease and their equivocal associations. Although the ultimate remedy for most patients is regulation of their body weight and diet, pharmacological therapies are still needed. The ideal drug for treating NASH should possess an assortment of characteristics, in that it should be able to reduce the hepatic fat content and also exert anti-inflammatory and antifibrotic effects [7]. Therefore, patients with NASH usually need to be prescribed several drugs for anti-obesity, anti-fatty liver, insulin receptor-sensitizing, anti-inflammatory, and antioxidative purposes [8]. However, taking a cocktail of drugs inevitably has latent side effects that limit its use. Therefore, herbal medicines, which mainly comprise crude extracts of medicinal plants, are used as conventional remedies globally. Although their overall chemical profiles are unknown and their mechanisms are unclear, the therapeutic effects and safety of medicinal plants in humans have been verified through their long-term use [9, 10]. Through their cocktail effects from a mixture of innate herbal compounds, the multipurpose therapeutic effects of herbal medicine on human diseases have been well documented. Considering their complex potencies, we hypothesized that the efficacy of herbal medicines in NASH may be evaluated suitably.

*Lysimachia vulgaris* var. *davurica* (LV), also known as the yellow loosestrife, is a medicinal plant that is well known for treating ulcers, fever, inflammation, and diarrhea [11]. Recent findings have revealed that LV also has antifungal, antibacterial, antitumor, and antioxidative activities [12, 13]. Moreover, LV is a non-toxic, edible plant that grows in Europe, North America, and Asian countries and is registered as a food ingredient in the Korean Food Standards Codex [14]. In a previous study, our research team found that LV is effective in a mouse model of diet-induced obesity, where it induced positive changes in lipid metabolism-related proteins and genes [15]. Moreover, we found some studies showing that *Lysimachia christinae* (which belongs to the same genus as LV) increased the detoxification mechanisms in liver [16, 17]. Therefore, we hypothesized that LV could alleviate NASH by regulating lipid metabolism, and may mitigate oxidative stresses in the liver as well. Because of the lack of a proper therapeutic agent for NASH, we wanted to verify whether LV could be a novel therapeutic agent for treating NASH. Hence, in this study, we determined the efficacy of LV for the treatment of NASH, a disease with multiple symptoms and complex developmental mechanisms.

## Methods

### Preparation of an LV plant extract

An LV plant was collected from Pyeongchang, Gangwon-do, Korea in May of 2015. Plant samples were collected from a government-owned forest, permission was obtained in advance from the national forest center. The specimen was identified by Dr. Hyung Seok Kim, and a voucher specimen was deposited in the Korea Institute of Science and Technology (KIST) as specimen KIST-JSH-1505. The plant was dried with a cold-air dryer at 30 °C for 7 days. In total, 5.88 kg of the dried aerial parts was extracted three times with 99.5% ethanol using a reflux system for 4 h, and the ethanol was evaporated in an evaporator at 40 °C. The prepared LV extract was an extremely sticky liquid.

### Animal model design and animal experiments

Five-week-old C57BLKS/J lar-Lepr^db^/Lepr^db^ (*db*/*db*) mice from Japan SLC, Inc. (Shizuoka, Japan) were obtained by the Central Lab. Animal, Inc. (Seoul, Korea). After 1 week of acclimation, 22 mice were separated into four experimental groups according to their body weight. After the acclimation period, the MCS group was provided a diet supplemented with methionine and choline, whereas the other groups were fed a diet deficient in methionine and choline (MCD) (Research Diets, New Brunswick, NJ, USA) for 3 weeks. During the experimental period, the vehicle solution (0.5% carboxymethyl cellulose [CMC]) was provided to the MCS and MCD groups by oral gavage. The GFT group was administered elafibranor (GFT505) in 0.5% CMC solution at a dosage of 10 mg·kg^− 1^·day^− 1^. Similarly, the LYS group was administered LV extract at a dose of 100 mg·kg^− 1^·day^− 1^. After 3 weeks, the mice were administered a mixture of ketamine (80 mg/kg) and xylazine (12 mg/kg) via intraperitoneal injection and blood samples were collected from the heart. Mice were sacrificed by excessive blood collection during the anesthetized state and liver samples were collected. The animal experimental designs were approved by the Institutional Animal Care Use Committee of KIST (approval number KIST-2018-107; date of approval, December 28, 2018).

### Biochemical analysis

The serum levels of alanine aminotransferase (ALT) (Cat. K752), aspartate aminotransferase (AST) (Cat. K753), TGs (Cat. K622), total cholesterol (TC) (Cat. K603), lactate dehydrogenase (LDH), high density lipoprotein cholesterol (HDL-c) (Cat. K613), and low density lipoprotein cholesterol (LDL-c) (Cat. K752) were analyzed using kits from BioVision (Minneapolis, MN, USA), following the manufacturer’s recommended protocols. The alkaline phosphatase (ALP) levels in serum samples were analyzed using an assay kit from ANASPEC (Cat. AS-72146) (Fremont, CA, USA). The concentrations of TG, TC, malonaldehyde (MDA) (Cat. K719), and hydroxyproline (Cat. K226) in liver tissues were also measured using kits from BioVision (USA).

### Histological analysis

Liver tissues extracted from each mouse were fixed in 10% formalin solution and then embedded in paraffin to form paraffin blocks. These blocks were then cut into thin sections, some of which were stained with hematoxylin and eosin (H&E). The sections were also subjected to Sirius Red staining to observe collagen in the liver. To observe hepatic lipids, frozen optimum cutting temperature-embedded liver tissue specimens were prepared. The tissues were then sectioned with a cryostat and fixed in 4% formalin solution before staining with Oil Red O. Histological scoring of images after H&E staining was conducted as described by Kleiner et al. [6].

### Real-time quantitative polymerase chain reaction (qPCR) experiments

Total RNA was extracted from liver cells using the RNeasy Mini Kit (Qiagen, Hilden, Germany) and then reverse transcribed to complementary DNA (cDNA) using the PrimeScript 1st Strand cDNA Synthesis Kit (Takara Bio Inc., Kusatsu, Japan). To quantify the levels of specific mRNA, the cDNA was mixed with SYBR Green Master Mix (Hoffmann-La Roche Ltd., Schweiz, Switzerland), and the fluorescence after qPCR was analyzed with a Light Cycler 480 device (Hoffmann-La Roche, Ltd.) during amplification. The primer sequences used in this study were as follows: 5′- AGG TGT CCC AAA GAA GCT GTA-3′ (F), 5′-ATG TCT GGA CCC ATT CCT TCT-3′ (R), monocyte chemoattractant protein-1 (*MCP-1*); 5′-GCC TTG GTA GAG GTG ACT GAG-3′ (F), 5′-GAC CGG AGC TGA AAA GTT GTA-3′ (R), intercellular adhesion molecule 1 (*ICAM-1*); 5′-GTG GGG GAC GAA GCG CAG AG-3′ (F), 5′-GGC CTT AGG GTT CAG CGG CG-3′ (R), alpha-smooth muscle actin (*α-SMA*); 5′-GAC GCC ATC AAG GTC TAC TG-3′ (F), 5′-ACG GGA ATC CAT CGG TCA-3′ (R), collagen type-1 alpha 1 (*COL1A1*); 5′-GAG GAA TGG GTG GCT ATC CG-3′ (F), 5′-TTG CGT CCA TCA AAG CCT CT-3′ (R), collagen type-3 alpha 1 (*COL3A1*); and 5′-GCC CAA TAC GAC CAA ATC C-3′ (F), 5′-AGC CAC ATC GCT CAG ACA C-3′ (R), Glyceraldehyde 3-phosphate dehydrogenase (*GAPDH*).

### Western blot analysis

Liver samples were lysed with RIPA buffer containing a protease inhibitor cocktail (Sigma–Aldrich, St. Louis, MO, USA), after which the protein concentration of each lysate was measured. The protein concentration of each liver lysate was then equalized by dilution in an appropriate volume of RIPA buffer, after which the loading dye was added. Detection of chemiluminescence signals via western blot analysis was conducted using an LAS-3000 Bio Imaging System (Fuji Film Co., Tokyo, Japan), and the band density was measured using Image J software (NIH, Bethesda, MD, USA). As for primary antibodies, nuclear factor E2-related factor 2 (Nrf2) (Cat. sc-13,032), Heme oxygenase 1 (HO-1) (Cat. sc-390,991), matrix metalloproteinase 2 (MMP-2) (Cat. sc-13,595), and α-SMA (Cat. sc-53,142) were obtained from Santa Cruz Biotechnology (Dallas, TX, USA). The primary antibodies against transforming growth factor beta (TGFβ) (Cat. #3711), Smad2/3 (Cat. #5678), p-Smad2/3 (Cat. #8828S), and Smad4 (Cat. #38454) were purchased from Cell Signaling Technology (Danvers, MA, USA).

### High-performance liquid chromatography and time-of-flight tandem mass spectrometry

The chemical profile of the ethanol extract of LV was analyzed by high-performance liquid chromatography-tandem mass spectrometry (HPLC-MS/MS). The Phenomenex Kinetex C18 reversed phase column (2.1 mm × 150 mm, 1.7 μm) that can construct ultra-HPLC (UPLC) analysis conditions in any liquid chromatography machine was equipped onto the HPLC apparatus (UltiMate 3000; Thermo Fisher Scientific, Waltham, MA, USA). Solvent A (0.1% formic acid in water) and solvent B (0.1% formic acid in acetonitrile) were prepared, and the mobile gradient was as follows: 0–10 min, with an initial mobile gradient of A and B of 99:1; 15 min, 90:10; 30 min, 70:30; 40–41 min, 30:70; 41–44 min, 0:100; and 45–50 min, 99:1. The flow rate was 0.2 mL/min, the column temperature was 45 °C, and detection was carried out at 254 nm. After separation of the chemical compounds with HPLC, MS was performed using a Q-TOF 5600+ device (AB Sciex, Framingham, MA, USA) with electrospray ionization. The negative ionization mode was used for focusing on the flavonoids and phenolic compounds in the plant. The MS scan mode (50–1000 m/z) was set to full scan and information-dependent acquisition. The MS operation conditions were as follows: 4.5 kV capillary voltage; 400 °C gas temperature; and 600 L/h gas flow. For automatic MS/MS scanning of the abundant chemical compounds in the sample, the MS survey mode was used with auto switching collision-induced dissociation, and the collision energy range was − 35 ± 15 eV. The tentative chemical compounds were determined by comparison against the analyzed MS/MS spectra and mass spectral libraries like the Human Metabolome Database and National Institute of Standards and Technology.

### Statistical analysis

The data generated in this study are presented as the mean ± standard deviation. For statistical analysis, one-way analysis of variance and Duncan’s multiple range test were performed using the SPSS statistics program, version 25.0 (IBM, Inc., Armonk, NY, USA).

## Results

### Putative chemical compounds of LV

Over 20 peaks were observed in the high-performance liquid chromatography (HPLC) chromatogram of an LV extract at 254 nm, with the largest peak detected at 28.15 min [see additional file 1]. Ten putative chemical compounds were identified by comparing tandem mass spectrometry signals and HPLC retention times against those of mass spectral libraries. The profile of chemical compounds in the LV extract differed slightly from that reported previously, although chlorogenic acid, ferulic acid, and rutin were found as the major compounds in common between this study and the previous study [18].

### LV alleviated liver damage in NASH

**Fig. 1:**
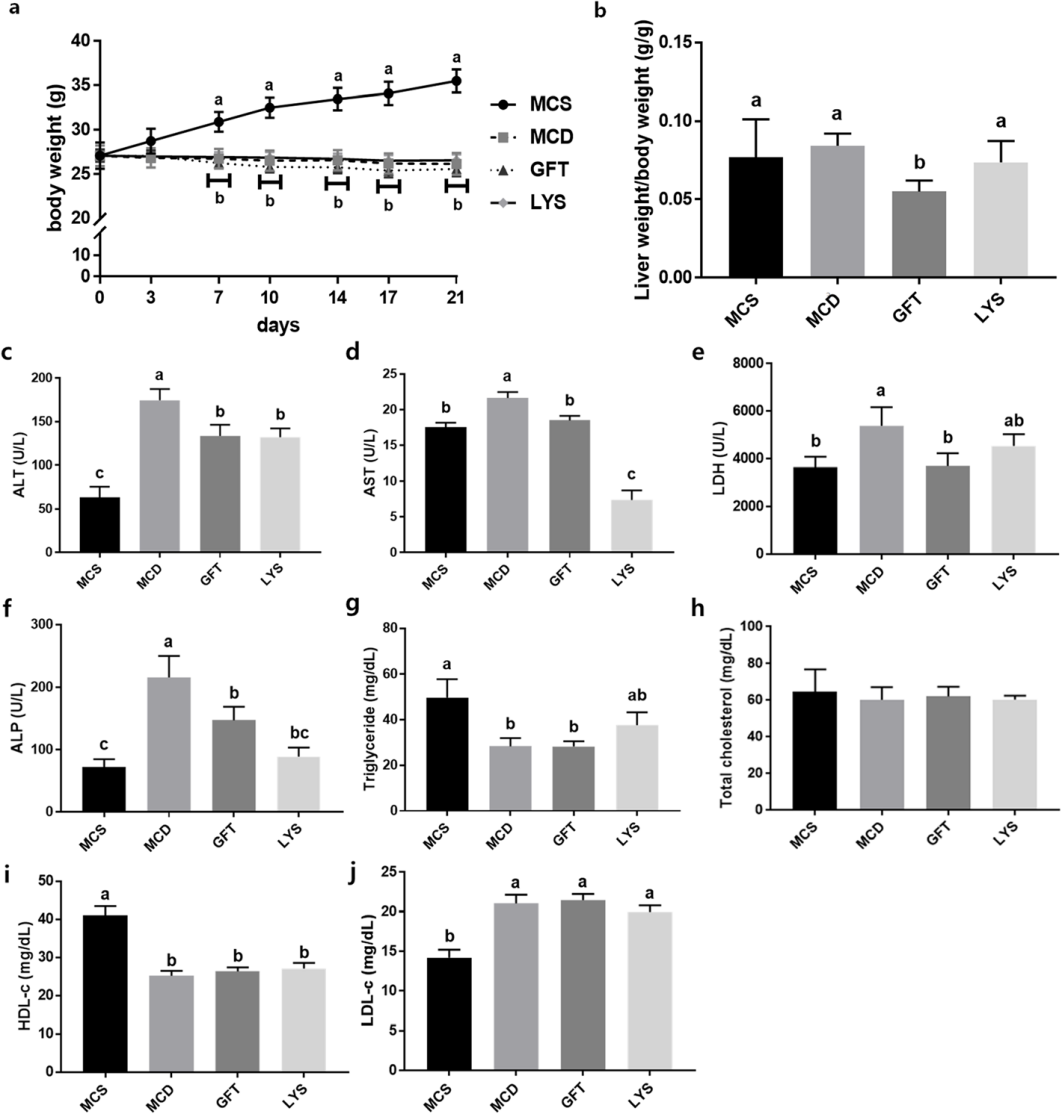
Body weights, liver weights, and biochemical indicators in the serum. Body weights were measured twice per week until day 21 (**a**). Liver weights were measured immediately after the mice were sacrificed; the liver weight/body weight ratio of each mouse is presented (**b**). The serum alanine aminotransferase (ALT) (**c**), aspartate aminotransferase (AST) (**d**), lactate dehydrogenase (LDH) (**e**), alkaline phosphatase (ALP), triglyceride (**g**), and total cholesterol (**h**), high density lipoprotein cholesterol (HDL-c) (**i**), low density lipoprotein cholesterol (LDL-c) (**j**) levels were measured. All data are presented as the mean ± SEM. One-way ANOVA and Duncan’s multiple range test were conducted. Different superscripts within groups indicate significant differences at *p* < 0.05

In contrast to the mice in the MCS group, mice in the MCD, GFT, and LYS groups all showed significantly reduced body weights (Fig. 1a). No significant difference in body weight was observed among the MCD diet-fed animals, with or without further treatment. However, mice in the positive-control group that was administered GFT505, showed significantly reduced liver weight/body weight ratios (Fig. 1b). Liver damage induced by NASH was examined by quantifying the ALT, AST, LDH, and ALP levels (Fig. 1c-f). All four enzyme were present at higher levels in the MCD group than in the MCS group, whereas AST, ALT, and ALP levels were significantly lower in the GFT and LYS groups (*p* < 0.05). Additionally, LDH levels in the LYS group was significantly decreased compare with MCD group. Meanwhile, the serum TG, TC, HDL-c, and LDL-c levels did not vary among the MCD diet-fed groups, and the serum TC levels were almost the same among all groups (Fig. 1g-j). This absence of changes in the serum TG and cholesterol levels in the MCD diet-fed *db*/*db* mouse model agreed with the previous observations [19, 20].

### LV reduced lipid accumulation and inflammation, and upregulated antioxidative proteins in the liver

**Fig. 2:**
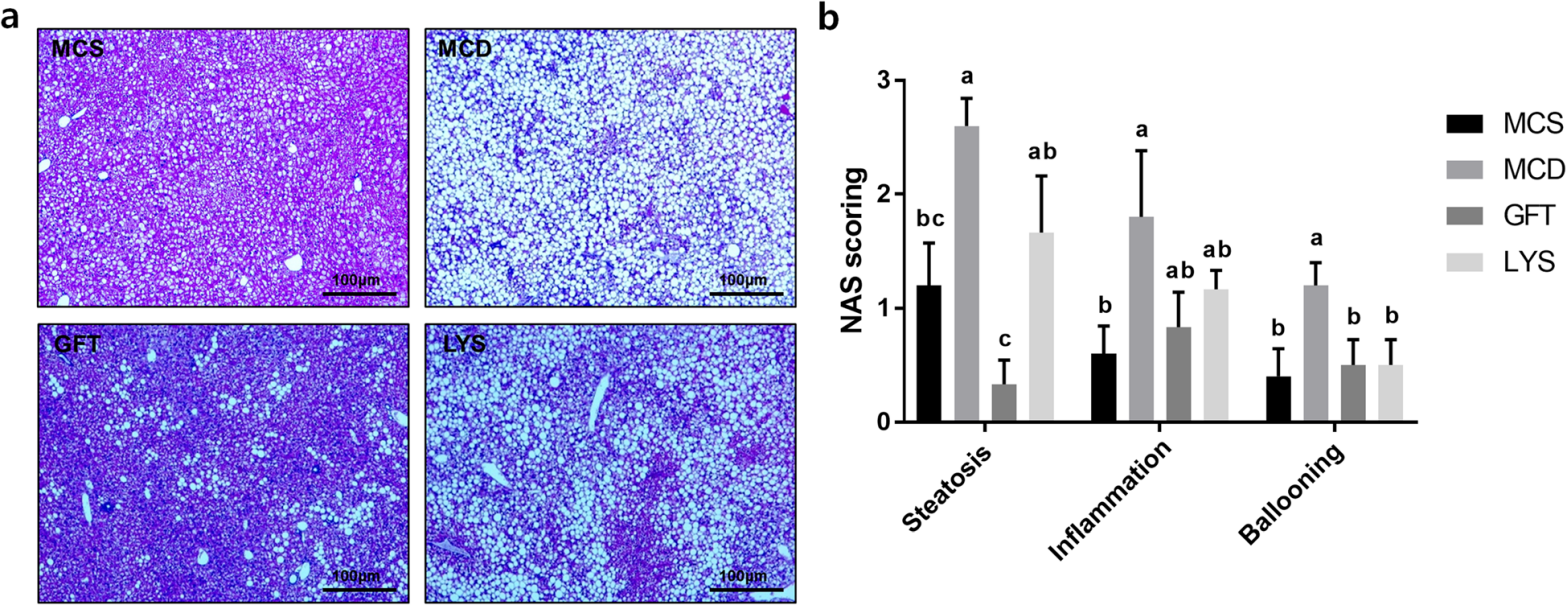
*Lysimachia vulgaris* var. *davurica*. (LV) ameliorated NASH pathogenesis. The liver tissues were stained with hematoxylin and eosin (**a**) and evaluated in terms of histopathological scores with NAS scoring (**b**). All data are presented as the mean ± SEM. One-way ANOVA and Duncan’s multiple range test were conducted. Different superscripts within groups indicate significant differences at *p* < 0.05

**Fig. 3:**
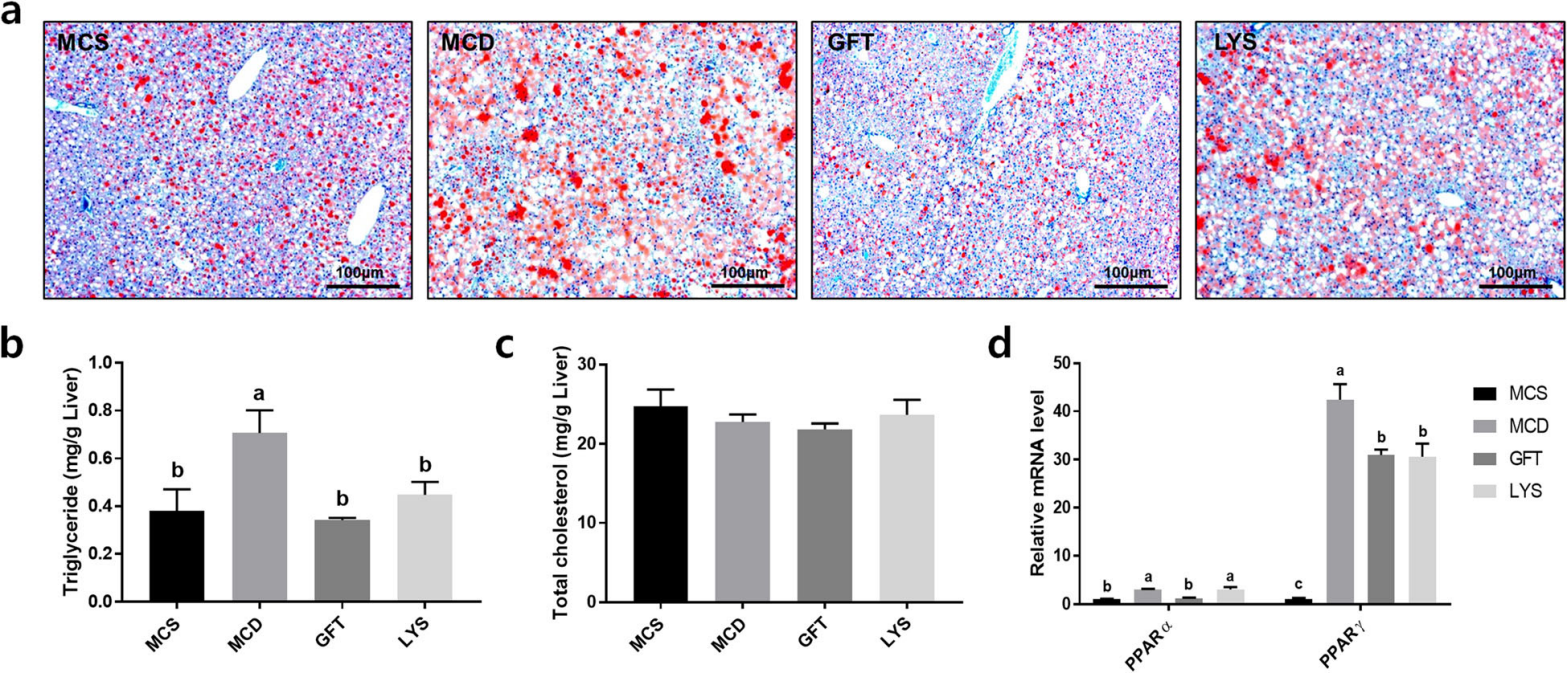
LV administration mitigated lipid accumulation in liver. Images of liver tissue sections stained with Oil Red O (**a**) and triglyceride (**b**), total cholesterol levels (**c**), and relative mRNA expressions of peroxisome proliferator-activated receptor-alpha (PPAR) α and γ (**d**) in liver tissues. All data are presented as the mean ± SEM. One-way ANOVA and Duncan’s multiple range test were conducted. Different superscripts within groups indicate significant differences at *p* < 0.05

Figure 2a shows changes in the liver morphology, as determined by H&E staining. The unstained white round spots in the image, which are supposed to be lipid droplets, were larger in the liver tissues of MCD mice than the MCS mice. However, lipid droplets seemed to be highly diminished in liver tissues from mice in the GFT group. Histological scoring (Fig. 2b) was conducted using images of H&E staining, and the GFT group showed significantly reduced lipid accumulation in their liver tissues. The LYS group did not show significant alleviation of lipid accumulation; however, anomalous ballooning of hepatocytes was significantly reduced similar to that in the GFT group. Oil Red O staining was performed to clearly visualize lipid accumulation (Fig. 3a). Similar to H&E staining, Oil Red O staining demonstrated that the MCD group had larger lipid droplets over a larger area compared to the MCS group. Administering the LV extract seemed to have reduced the number of lipid droplets, and the GFT group seemed to highly reduce lipid accumulation, compared to the MCS group. The TG levels in liver tissues from the LYS and GFT groups decreased significantly as shown in Fig. 3b. However, no changes were observed in the liver TC levels, similar to the serum TC levels (Fig. 3c). Meanwhile, the mRNA levels of PPAR a and PPARr were determined as a key mediator of lipid homeostasis. (Fig. 3d). PPARα level was significantly increased in the MCD group; however, this level was significantly lowered by GFT treatment (*p* < 0.05). Likewise, PPARγ was increased by almost 40 times in the MCD group, whereas the GFT and LYS groups showed significantly decreased PPARγ expression (*p* < 0.05).

**Fig. 4:**
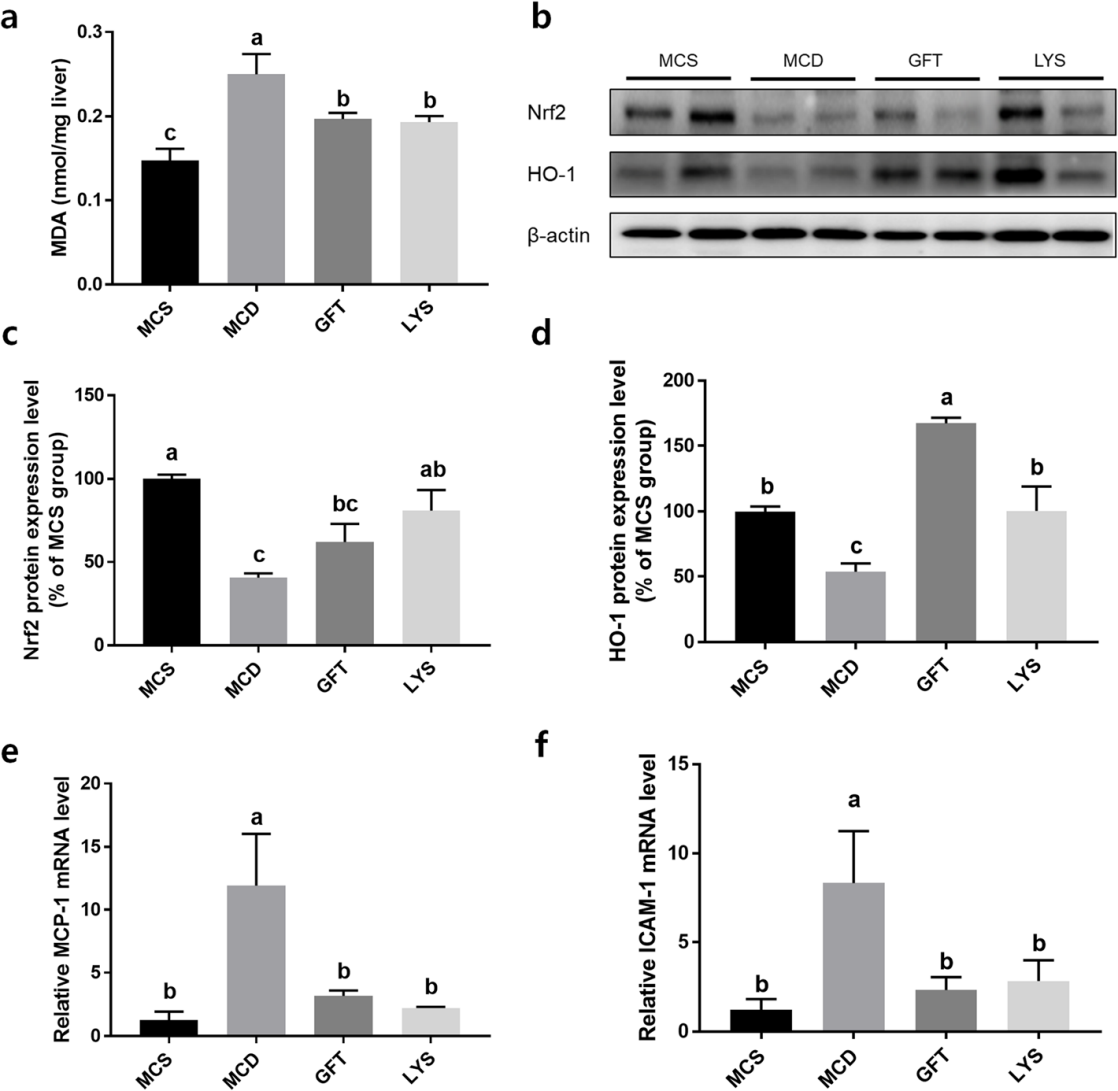
LV extract elevated expression levels of anti-oxidative related proteins and inflammatory related mRNAs. The malonaldehyde (MDA) contents of liver tissues were analyzed (**a**). The bands were imaged after western blot analysis (**b**). The expression levels of the nuclear factor-E2 related factor 2 (Nrf2) (**c**) and heme oxygenase-1 (HO-1) (**d**) proteins were quantified by comparing their relative band densities to that of β-actin, using ImageJ software. The relative mRNA-expression levels of the monocyte chemoattractant protein-1 (*MCP-1*) (**e**) and intercellular adhesion molecule 1 (*ICAM-1*) genes (**f**) were quantified by performing quantitative polymerase chain reaction (qPCR) experiments. All data are presented as the mean ± SEM. One-way ANOVA and Duncan’s multiple range test were conducted. Different superscripts within groups indicate significant differences at *p* < 0.05

The MDA contents of liver tissues were analyzed for checking oxidative stress in the liver (Fig. 4a). The MDA content was significantly increased in the MCD group compared with that in the MCS group, whereas administration of GFT and LYS resulted in significantly decreased oxidative stress in the liver tissues (*p* < 0.05). The protein-expression levels of Nrf2 and HO-1 were significantly lower in the MCD group than in the MCS group (*p* < 0.05; Fig. 4b-d). In contrast, treatment with GFT505 and the LV extract increased the protein-expression levels of Nrf2 and HO-1. Oxidative stress in cell systems can lead to inflammatory responses. Therefore, we anticipated that the LV extract could reduce inflammatory reactions by protecting cells against reactive oxygen species (ROS) induced due to NASH. To determine the extent of the inflammatory signaling in NASH, the gene-expression levels of *MCP-1* and *ICAM-1* were determined (Fig. 4e-f). Only the MCD group showed markedly higher expression levels of these two inflammation-related genes, whereas they were significantly downregulated by the LV extract and GFT505 (*p* < 0.05).

### LV alleviated NASH-related liver fibrosis and downregulated TGFβ/Smad-dependent signaling

**Fig. 5:**
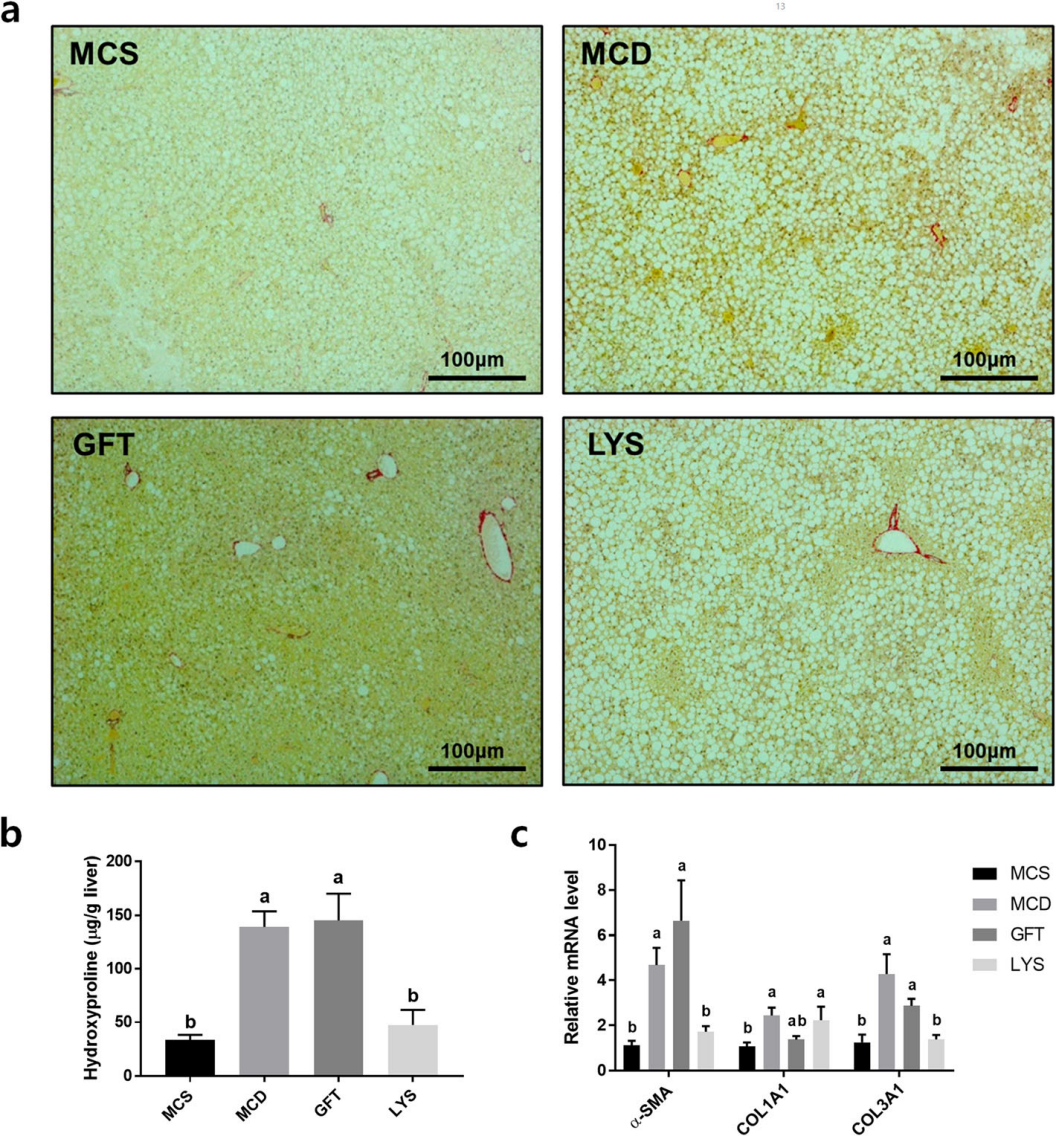
LV extract alleviated liver fibrosis in NASH mice model. The mouse liver tissues were sectioned and stained with Sirius Red (**a**). The liver hydroxyproline content was measured with a commercial kit (**b**). The expression levels of genes related to liver fibrosis were determined by qPCR, including alpha smooth muscle actin (*α-SMA*), collagen, type-1, alpha 1 (*COL1A1*), and collagen, type-3, alpha 1 (*COL3A1*) (**c**). All data are presented as the mean ± SEM. One-way ANOVA and Duncan’s multiple range test were conducted. Different superscripts within groups indicate significant differences at *p* < 0.05

**Fig. 6:**
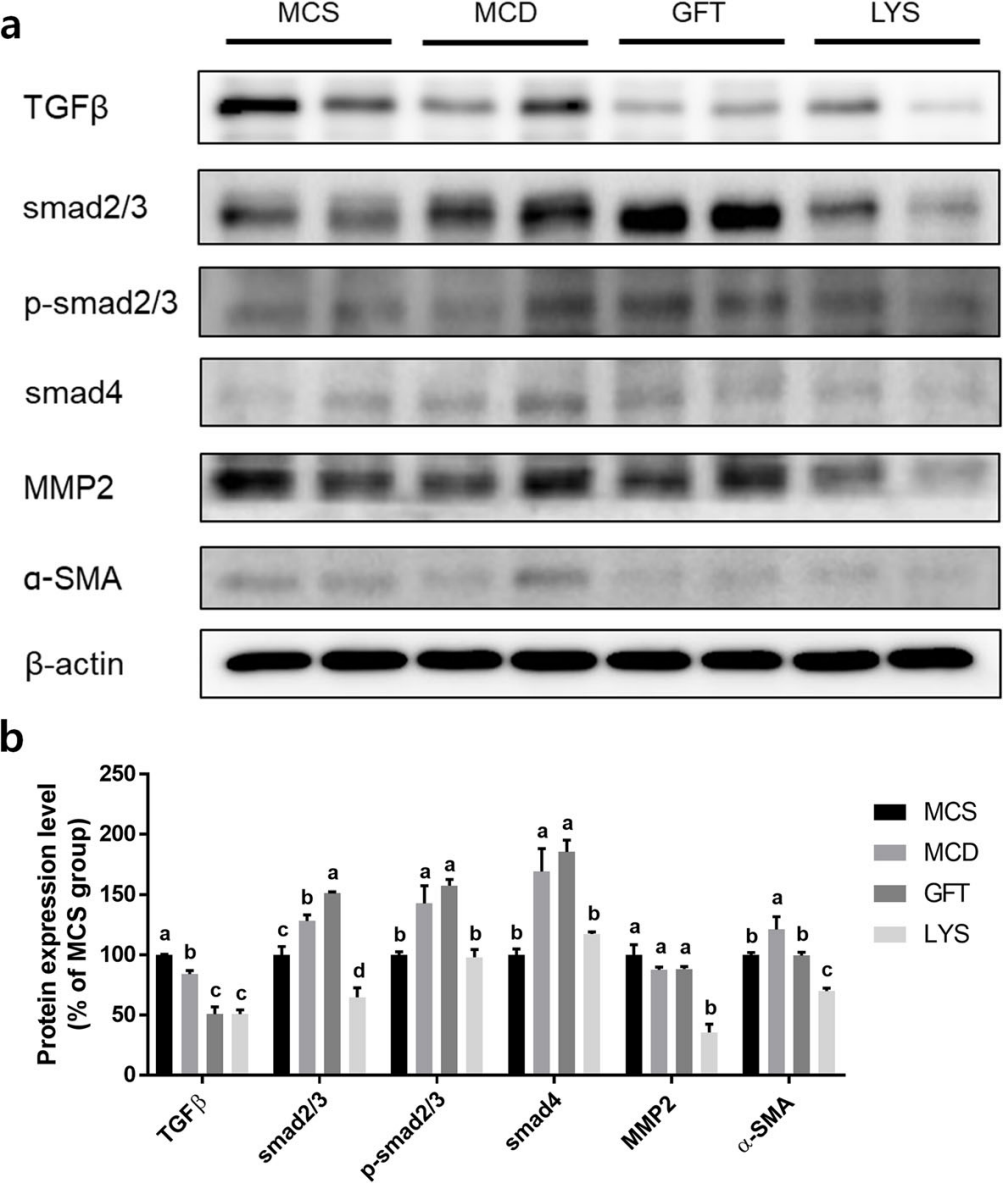
LV extract downregulated the TGFβ and Smad-dependent pathway in NASH mice. Protein-expression levels of TGFβ and transcriptional factors of fibrotic proteins were detected in liver tissues from mice with diet-induced nonalcoholic steatohepatitis (**a**). The relative protein-expression levels of TGFβ, Smad2/3, p-Smad2/3, Smad4, MMP2, and α-SMA (**b**) were determined. All data are presented as the mean ± SEM. One-way ANOVA and Duncan’s multiple range test were conducted. Different superscripts within groups indicate significant differences at *p* < 0.05

Sirius Red was used to stain the fibrotic regions in liver tissues, which appeared as yellow areas under the microscope (Fig. 5a), and these fibrotic regions in the liver were quantified by measuring the amounts of hydroxyproline in the tissues (Fig. 5b). Liver fibrosis decreased in the LYS group; however, interestingly, GFT505 did not ameliorate liver fibrosis. The GFT group presented high levels of hydroxyproline and in amounts similar to those in the MCD group. In contrast, the LYS group showed significantly reduced hydroxyproline levels. In addition, the mRNA expression levels of fibrogenesis-related genes decreased after treatment with LV extract (Fig. 5c). Moreover, we found that the levels of TGFβ and Smad signaling-related protein (TGF-β1, Smad2/3, p-Smad2/3, and Smad4), one of the key mechanisms regulating the expression of fibrotic genes, was significantly decreased in the LYS group compared with MCD (Fig. 6).

## Discussion

The MCD diet is a well-established experimental model for inducing severe NAFLD and NASH in animals. The liver is the major organ of lipogenesis in the body, being the site for both fatty acid uptake and de novo fatty acid synthesis. After hepatic lipogenesis, the liver secretes lipids into the portal vein, mostly in the form of VLDL, which consists mainly of apolipoprotein B and phosphatidyl choline (PC) [21]. Choline and methionine are precursors of PC; hence, the MCD diet causes a lack of PC in mice and consequently blocks the secretion of synthesized TGs from the liver to the blood [19]. Hence, mice fed the MCD diet accumulate excess lipids in the liver, which causes severe NAFLD. Moreover, the accumulated lipids undergo beta-oxidation; with ensuing excessive oxidative stress, this causes inflammation of hepatocytes and ultimately NASH [22]. However, there are drawbacks to mimicking human NAFLD in a mouse model with only the MCD diet. Most human patients with NASH and NAFLD have underlying obesity, whereas mice with MCD diet-induced NASH show a loss in body weight. Thus, genetically modified mice should be used to mimic the human disease. For example, the *db*/*db* mouse is a genetically modified animal that is used to study obesity and diabetes, and upon MCD diet administration, their pathogenic progression from obesity to NAFLD and NASH is similar to that of humans [23]. Therefore, *db*/*db* mice fed an MCD diet were used as the NASH model in this study. The severity of NASH in mice was evaluated with NAS scoring. The results revealed that ballooning significantly reduced in GFT and LYS groups (*p* < 0.05). Moreover, the values of other NAS factors (steatosis and inflammation) decreased, albeit without statistical difference. Therefore, we thought that LV could relieve NASH pathogenesis; however, the GFT group showed high efficacy in NASH treatment. Notably, GFT505 (a dual PPARα/δ agonist) has been demonstrated to improve lipid and glucose homeostasis in patients with abdominal obesity [24]. Moreover, PPARs are nuclear receptors that play key roles in regulating lipid metabolism; these are also related to inflammation, cellular growth, and cell differentiation [25]. GFT505 caused downregulation of mRNA levels of *PPARα* and *PPARγ* in liver tissues, and the LYS group also showed decreased *PPARγ* gene expressions. As a result, the GFT and LYS group showed significant mitigation of lipid accumulation in the liver compared to the MCD group.

Excessive accumulation of lipids in the liver causes inflammatory reactions, where oxidative stress may result from lipid oxidation. The Nrf2 and antioxidant-response element (ARE) pathway is one of the major detoxification pathways that enhances antioxidative responses [26]. Nrf2 belongs to the cap ‘n’ collar (CNC) family of basic-region leucine zipper (b-Zip) motifs, and Nrf2 and ARE regulate the phase II family of enzymes [27]. Among the CNC family members, Nrf2 is considered a key regulator that induces antioxidant-responsive genes by interacting with ARE [28]. *HO-1*, which is an antioxidant-responsive gene regulated by Nrf2, degrades heme to generate ferrous ion, CO, and biliverdin, all of which are related to the reduction of ROS [29]. In addition, few studies have reported the antioxidative activity of LV extract [12, 13]. Consistent with these findings, we found that liver Nrf2 and HO-1 protein levels in LYS group were significantly increased, indicating that the extract regulates the Nrf2 activator within the cellular system. In addition, many lines of evidence indicate that Nrf2 and HO-1 can prevent lipid oxidation in vivo and that Nrf2 activation is sufficient to attenuate hepatic lipid accumulation in mice with alcohol-induced fatty liver [30, 31]. Therefore, attenuation of lipid accumulation by the LV extract could be related to Nrf2 activation; however, further studies are needed to verify this possibility.

Liver fibrosis, which results from the replacement of hepatocytes with fibrotic cells during NASH pathogenesis, causes irreparable damage to the liver and loss of liver function. During liver cirrhosis, fibrous scars form along with the generation of myofibroblasts, the main sources of which are hepatic stellate cells (HSCs) [32]. In the normal liver, the major role of HSCs is in storing retinyl esters, a stored form of vitamin A [33]. However, HSCs are activated and transformed into myofibroblasts upon liver injury, and the expression levels of the related genes *α-SMA*, *COL1A1*, and *COL3A1* can increase [34]. In particular, the expression level of *α-SMA* is a reliable marker of activated HSCs, and is also strongly correlated with myofibroblast formation [35, 36]. The mRNA-expression level of *α-SMA* was the highest in the GFT group, indicating that although GFT505 could attenuate lipid accumulation, oxidative stress, and inflammatory reactions in NASH, its recovery of damaged hepatocytes was not very effective. In contrast, the LYS group showed decreased levels of fibrosis-related genes. Meanwhile, as one of the key activator of HSCs in NASH pathogenesis, the inflammatory responses induced by Kupffer cells (KCs) are highly associated with NASH [37]. KCs are the resident macrophages in hepatic tissues, and produce pro-inflammatory cytokines and chemokines when NASH occurs [38, 39]. Thus, KC activation is linked with the exacerbation of liver fibrosis in NASH pathogenesis. Although specific markers that can prove KC activation have not been determined in this study, we found that the contents of crucial chemokines (MCP-1 and ICAM-1) are decreased when LV and GFT are administered. Furthermore, we noted TGFβ as a mediator of inflammation and liver fibrosis mechanisms [38].

Substantial evidence indicates that TGFβ and Smad-dependent downstream signaling are key regulatory factors in liver fibrosis [40, 41]. TGFβ receptor activation causes Smad2/3 phosphorylation and binding with Smad4, and this oligomer induces the transcription of fibrogenesis-related genes after it translocates to the nucleus [42]. TGFβ represents a predominant and central mediator of fibrosis in many organs in addition to the liver; therefore, it has been the target of therapeutic agents against fibrosis [43]. These protein-expression changes resulted in diminished α-SMA expression, a final product. In addition, the LV extract also significantly decreased (*p* < 0.05) the expression of MMP-2. The inactive form of TGFβ complexes with the latency TGF-binding protein (LTBP), and after dissociating from MMP-2, the active form of TGFβ can be released [44]. Hence, it is surmised that the LV extract can ameliorate liver fibrosis by regulating an MMP-2-related pathway, although further studies are needed to verify this mechanism.

## Conclusions

In summary, we administered an LV extract to mice with MCD diet-induced NASH. The histopathological grades of NASH (based on the morphologies of liver sections) were ameliorated by LV supplementation, and lipid accumulation in the liver tissue also decreased. Moreover, treatment with the LV extract upregulated Nrf2, which may have improved the antioxidative and anti-inflammatory activities in the liver. Additionally, the LV extract strongly alleviated liver fibrosis by downregulating *α-SMA* and *COL3A1*. Mice fed the LV extract also showed decreased levels of proteins related to TGFβ and Smad signaling, which act upstream of fibrotic gene expression. Therefore, the potency of the LV extract in relieving liver fibrosis may result from blocking TGFβ and Smad signaling. In conclusion, the LV extract ameliorated NASH by alleviating lipid accumulation, oxidative stress, inflammation, and LV extract successfully relieved liver fibrosis. In particular, LV extract ameliorated liver fibrosis effectually in comparison with a dual PPARα/δ agonist (GFT 505). These findings indicate many advantages for using the LV extract as a therapeutic agent against NASH.

## Supplementary Information

**Additional file 1.** HPLC chromatogram at 254 nm and mass spectra of the LV ethanol extract.

**Additional file 2.** The uncropped membrane images of western blotting analysis.

## Data Availability

Not applicable.

## Abbreviations

NASH: Nonalcoholic steatohepatitis
LV: *Lysimachia vulgaris* var. davurica
db/db: C57BLKS/J lar-Lepr^db^/Lepr^db^
MCD: Methionine-choline deficient
NAFLD: Nonalcoholic fatty liver disease
TGs: Triglycerides
VLDLs: Very-low-density lipoproteins
CMC: Carboxymethyl cellulose
ALT: Alanine aminotransferase
ALP: Alkaline phosphatase
AST: Aspartate aminotransferase
TC: Total cholesterol
LDH: Lactate dehydrogenase
H&E: Hematoxylin and eosin
qPCR: Real-time quantitative polymerase chain reaction
cDNA: Complementary DNA
HPLC: High-performance liquid chromatography
PPARɑ: Peroxisome proliferator-activated receptor-alpha
Nrf2: Nuclear factor E2-related factor 2
ROS: Reactive oxygen species
MCP-1: Monocyte chemoattractant protein-1
ICAM-1: Intercellular adhesion molecule 1
TGFβ: Transforming growth factor beta
PC: Phosphatidyl choline
CNC: Cap ‘n’ collar
b-Zip: Basic-region leucine zipper
HSCs: Hepatic stellate cells
α-SMA: Alpha-smooth muscle actin
COL1A1: Collagen type-1 alpha 1
COL3A1: Collagen type-3 alpha 1
KC: Kupffer cell
MMP-2: Matrix metalloproteinase 2
LTBP: Latency TGF-binding protein
