# Supplementary material for: Yellow loosestrife (Lysimachia vulgaris var. davurica) ameliorates liver fibrosis in db/db mice with methionine- and choline-deficient diet-induced nonalcoholic steatohepatitis
Source: BMC Complement Med Ther. 2021 Jan 25;21:44. doi: 10.1186/s12906-021-03212-6 (PMC7836176; doi:10.1186/s12906-021-03212-6)
Supplement: Supplementary file 1 — Additional file 1. HPLC chromatogram at 254 nm and mass spectra of the LV ethanol extract. [file 12906_2021_3212_MOESM1_ESM.docx]

Additional file 1. HPLC chromatogram at 254 nm and mass spectra of the *Lysimachia vulgaris* var. *davurica* ethanol extract. Putative chemical compounds were retrieved from MS/MS library using q-TOF MS/MS analysis.





| No. | T_r_ (min) | [M-H]^-^ (m/z) | Fragment ions (m/z) | Putative chemical compound |
| --- | --- | --- | --- | --- |
| 1 | 17.78 | 353.088 | 192, 161 | Neochlorogenic acid |
| 2 | 20.57 | 307.141 | 161, 119, 89, 71, 59 | Phaseolic acid |
| 3 | 21.99 | 367.105 | 179, 135 | 5-O-Caffeoylquinic acid methyl ester |
| 4 | 23.29 | 609.147 | 301, 271, 255 | Rutin |
| 5 | 23.71 | 463.089 | 302, 85 | Spiraeoside |
| 6 | 24.37 | 309.063 | 162, 129 | Bergenin |
| 7 | 24.98 | 193.051 | 133, 106 | Ferulic acid |
| 8 | 27.99 | 299.188 | 201, 184 | Kessyl glycol |
| 9 | 35.50 | 309.208 | 291, 239, 221, 183, 171 | Sterebin A |
| 10 | 36.02 | 262.193 | 221, 203 | Santalyl acetate |
