# Supplementary material for: Yellow loosestrife (Lysimachia vulgaris var. davurica) ameliorates liver fibrosis in db/db mice with methionine- and choline-deficient diet-induced nonalcoholic steatohepatitis
Source: BMC Complement Med Ther. 2021 Jan 25;21:44. doi: 10.1186/s12906-021-03212-6 (PMC7836176; doi:10.1186/s12906-021-03212-6)
Supplement: Supplementary file 2 — Additional file 2. The uncropped membrane images of western blotting analysis. [file 12906_2021_3212_MOESM2_ESM.docx]

**Images of uncropped gels**

Nrf2 (Fig. 4a)


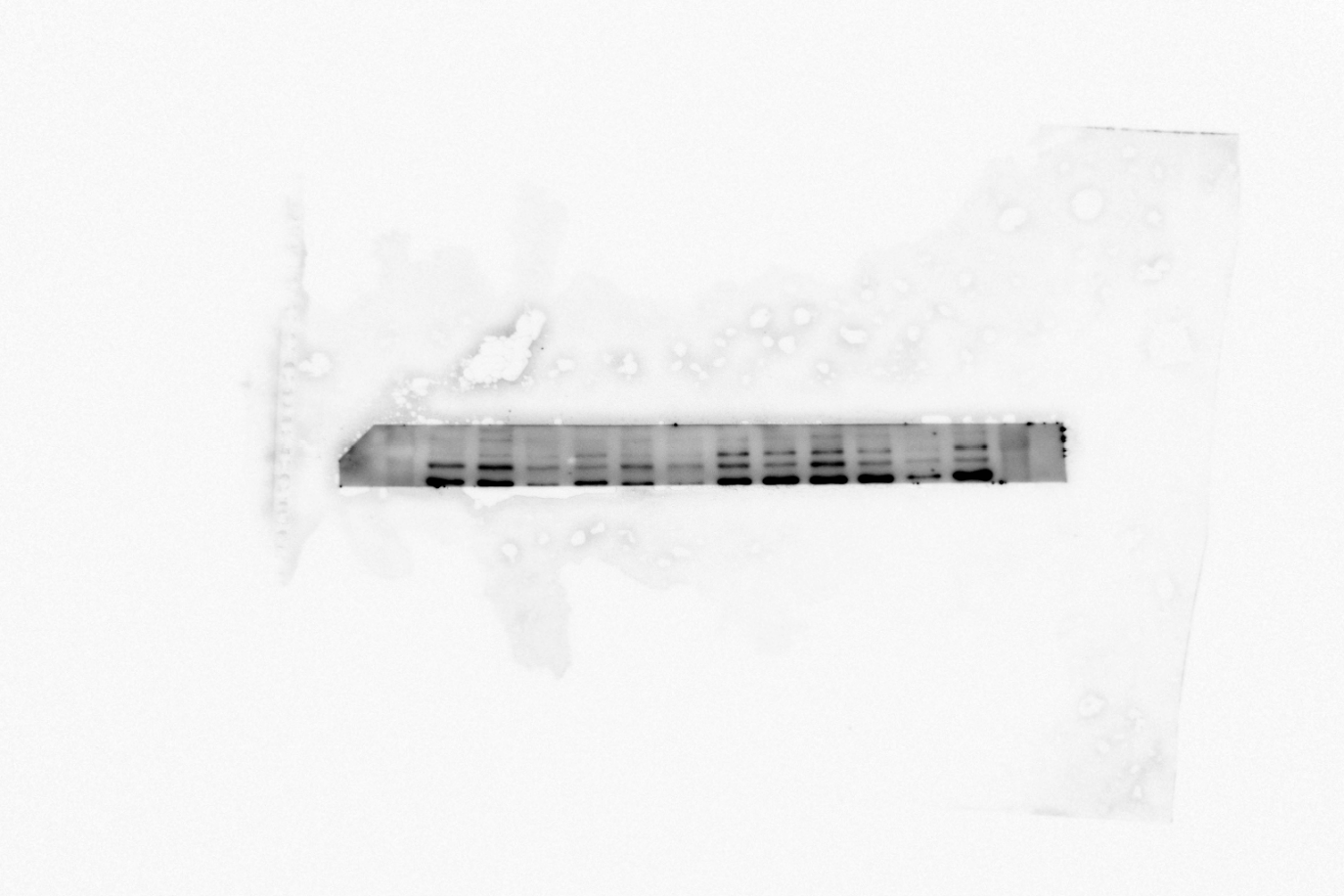


100 kDa

HO-1 (Fig. 4a)


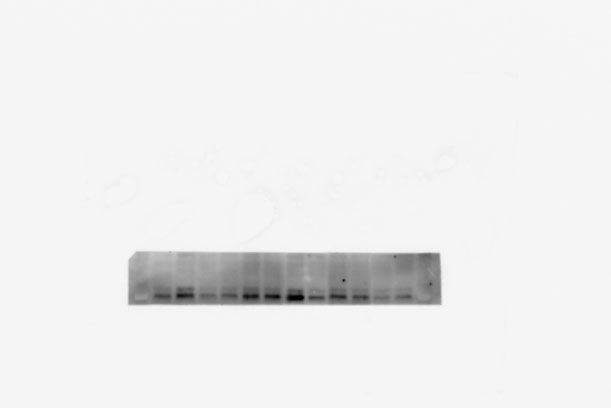


32 kDa

β-actin (Fig. 4a)


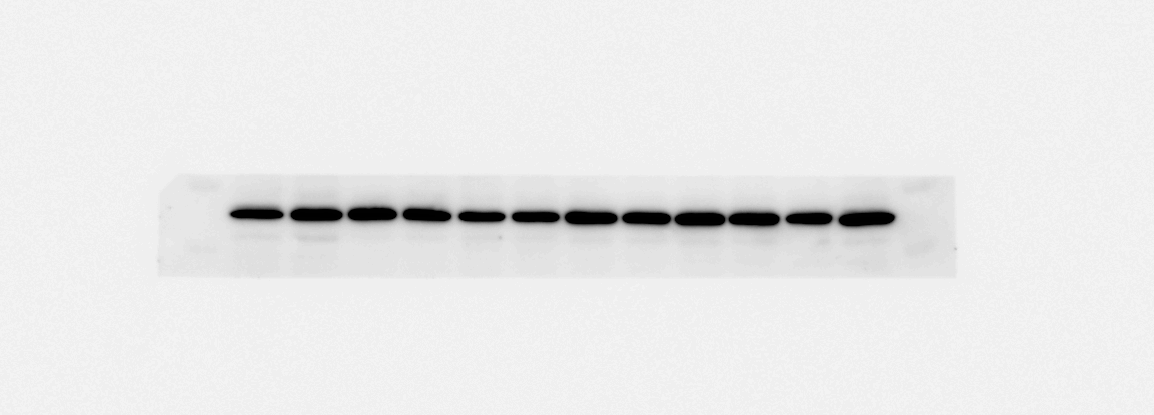


43 kDa

TGF β (Fig. 6a)


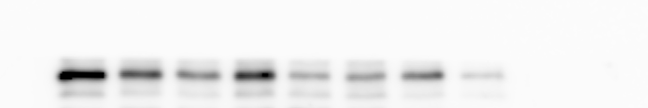


25 kDa

smad2/3 (Fig. 6a)


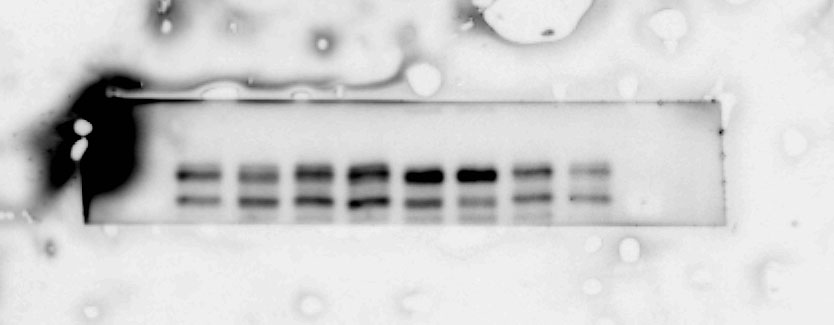


60 kDa

p-smad2/3 (Fig. 6a)


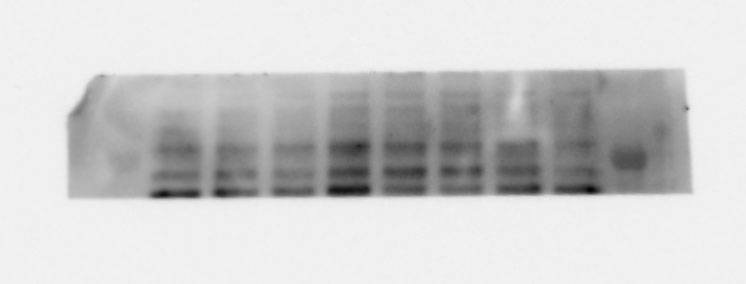


60 kDa

Smad4 (Fig. 6a)


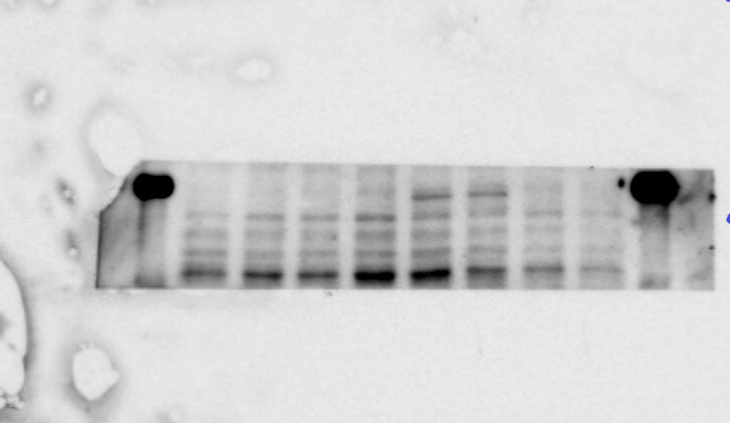


70 kDa

MMP2 (Fig. 6a)


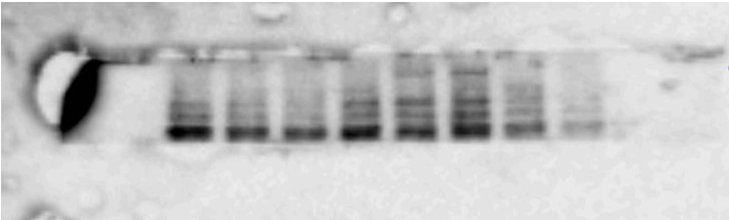


68 kDa

α-SMA (Fig. 6a)


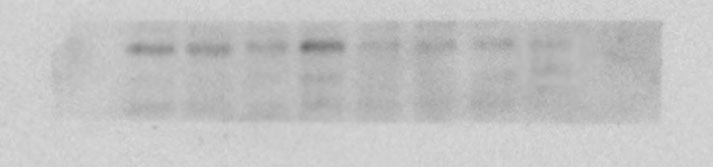


43 kDa

β-actin (Fig. 6a)


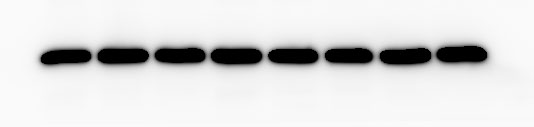


43 kDa
